# Tortuous Carotid Arteries and Their Clinical Implications: A Report of Two Cases

**DOI:** 10.7759/cureus.36953

**Published:** 2023-03-31

**Authors:** Anisa Raidah, Nolberto Jaramillo, Giuseppe Serena, Steven Lev, Anantha Ramanathan

**Affiliations:** 1 Surgery, New York Institute of Technology College of Osteopathic Medicine, Old Westbury, USA; 2 Surgery, Nassau University Medical Center, East Meadow, USA; 3 Neuroradiology, Nassau University Medical Center, East Meadow, USA

**Keywords:** carotid artery kinking, tortuous carotid artery, congenital vascular anomaly, common carotid artery, internal carotid artery

## Abstract

The tortuous carotid artery is a rare anatomic abnormality defined as vascular elongation leading to an altered course. It can be discovered incidentally or have clinically significant manifestations. The most common location is the internal carotid artery or, less commonly, the common carotid artery. Bilateral tortuous carotid arteries can also occur, leading to "kissing carotids" where the carotid arteries are juxtaposed. We describe two cases of carotid artery tortuosity in patients with risk factors associated with its development. One case is of a 91-year-old female presenting with a cerebrovascular accident and an incidental finding of tortuosity of the right common carotid artery mimicking the appearance of "kissing carotids." The other case concerns a 66-year-old female with a symptomatic tortuous left internal carotid artery. This report aims to inform clinicians of the differences in the anatomical features, pathogenesis, and possible clinical implications of these variants.

## Introduction

Carotid artery tortuosity is an anatomic variant where a segment of the internal carotid artery (ICA) or common carotid artery (CCA) is elongated and redundant, presenting as coiling, looping, or kinking. When this phenomenon occurs bilaterally in the ICA, it is commonly referred to in the literature as retropharyngeal ICA or “kissing carotids,” where the carotid arteries course medially [1]. Although both are rare (incidence 18-34%), tortuosity of the ICA is more common. Previous studies have shown that these variants are often incidental findings. However, they may have clinical implications, including mass effects, ischemic symptoms, and an increased risk of procedural complications [2,3]. We present two rare cases of carotid artery tortuosity: the first is an asymptomatic tortuous right common carotid artery with the appearance of “kissing carotid arteries.” The second case is a symptomatic patient with a kinked left internal carotid artery. The cases presented in this report highlight the etiologies and clinical implications of these rare variants.

## Case presentation

Case 1

**Figure 1:**
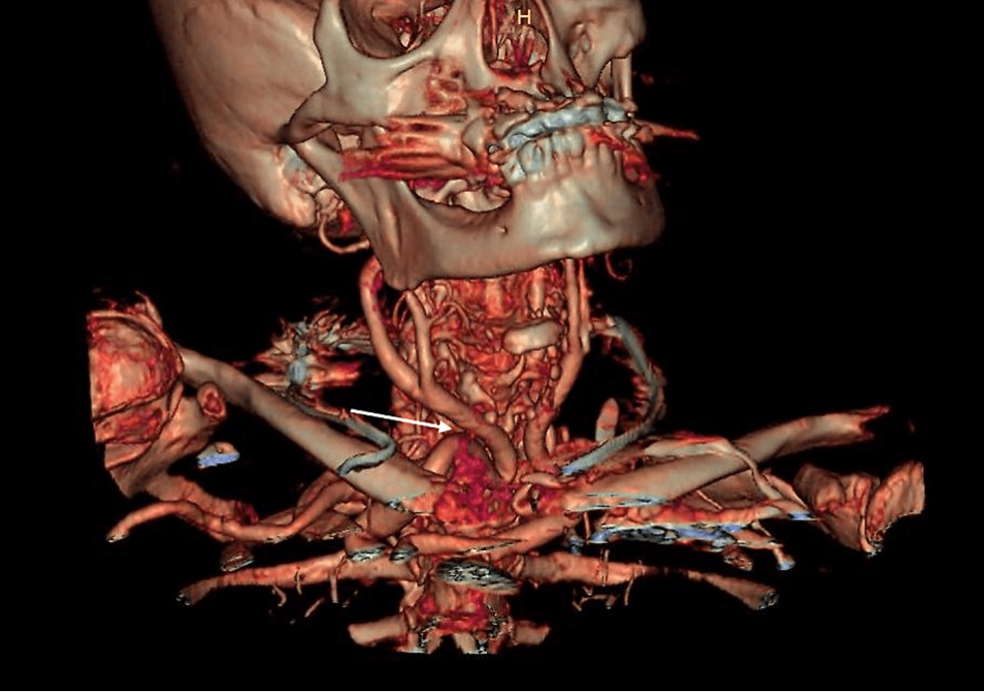
Tridimensional reconstruction of kissing carotids. The arrow points to the tortuous right common carotid artery that comes in close proximity to the left.

A 91-year-old female presented to the emergency department with left-sided neurological deficits, word-finding difficulty, and confusion. A review of her medical history showed right-sided cerebrovascular accident (CVA), hyperlipidemia, hypertension, atrial fibrillation, and atherosclerotic cardiovascular disease. Her National Institutes of Health (NIH) Stroke Scale score was 10, and she was admitted secondary to a hypertensive emergency. On physical examination, the patient was somnolent and difficult to rouse. Magnetic Resonance Imaging (MRI) revealed a left middle cerebral artery (MCA) infarct. Computed Tomography Angiography (CTA) of the head and neck demonstrated tapering and complete occlusion of the left internal carotid artery origin with significant plaque formation. The right CCA was tortuous and coiled, resulting in its proximity to the left CCA (Figure 1).

This incidental finding did not contribute to the patient's symptoms. The right internal and external carotid arteries resumed their ordinary course. The patient did not meet the criteria for thrombolysis due to being on anticoagulation, and neurosurgery was not pursued due to her age. Subsequently, the patient’s family elected to make her not resuscitate or intubate, and limited medical intervention status. After a six-week course in the hospital under palliative care, she eventually expired due to cardiopulmonary arrest. 

Case 2

**Figure 2:**
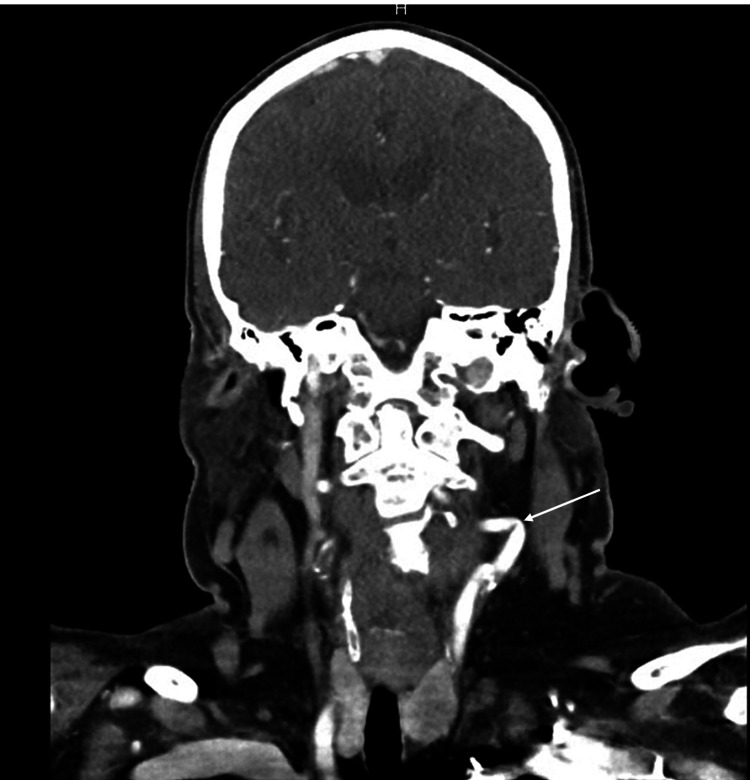
Computed tomography angiography of the head and neck demonstrating tortuosity of the left internal carotid artery.

A 66-year-old female presented to the emergency department following two episodes of dizziness and nausea associated with a sudden movement of the head. Her medical history was positive for CVA, end-stage renal disease (ESRD) on hemodialysis, hypertension, diabetes mellitus type 2, hyperlipidemia, and myocardial infarction with stent placement (on aspirin and ticagrelor). CTA of the head and neck showed high-grade stenosis of the distal left vertebral artery with significant kinking of the left internal carotid artery in the sagittal view of CTA (Figure 2).

**Figure 3:**
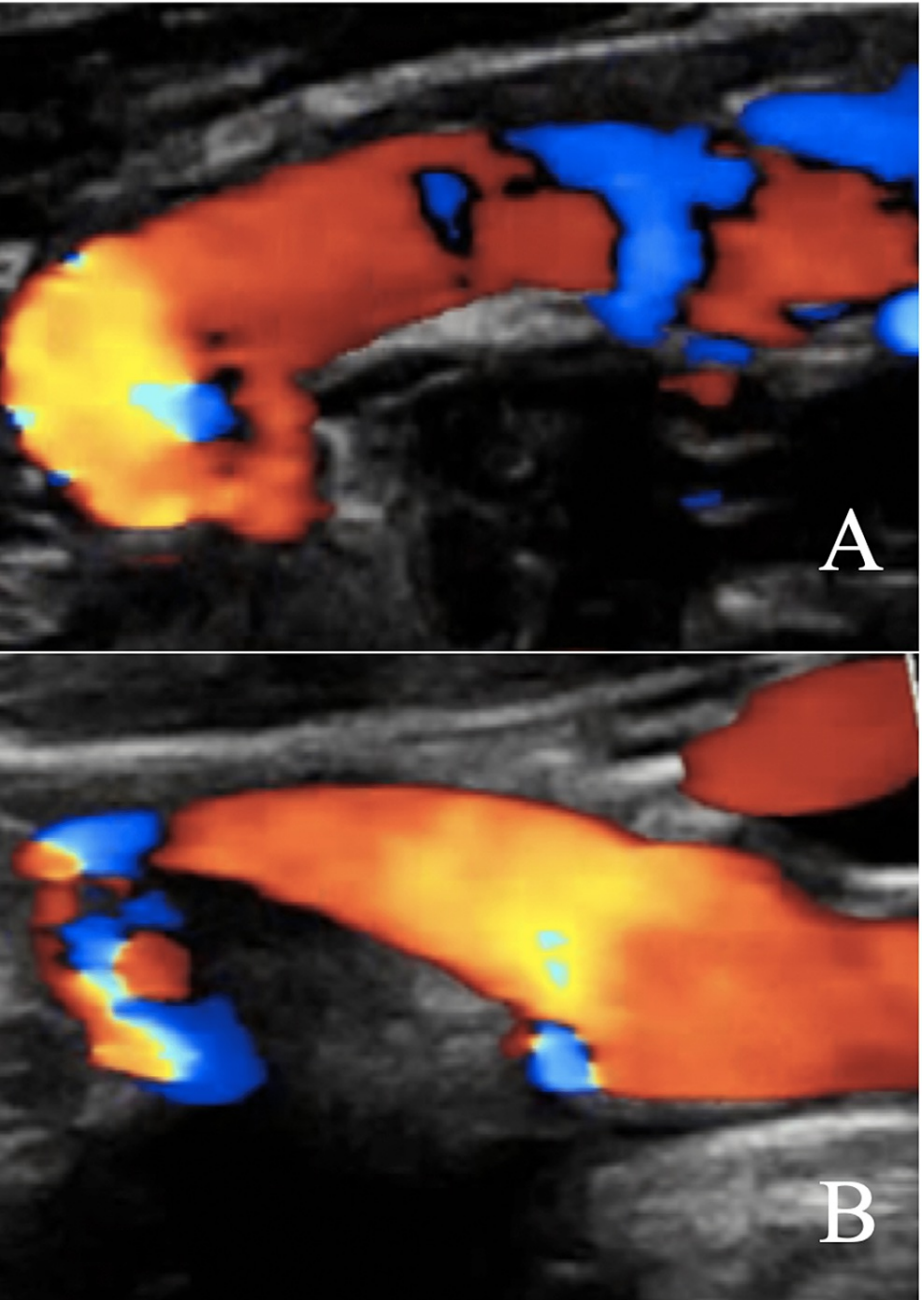
Duplex ultrasound of the left distal internal carotid artery demonstrating change of flow velocity and diameter based by head positioning. (A) Head straight (131 cm/second and 6 mm) (B) Head turned to the right (213 cm/second and 2.3 mm).

Duplex carotid ultrasound was performed with the head straight and then turned to the right, demonstrating an increase in peak systolic velocity (131 cm/second and 213 cm/second, respectively) and a decrease in the diameter of the vessel (6 mm and 2.3 mm, respectively) (Figure 3).

**Figure 4:**
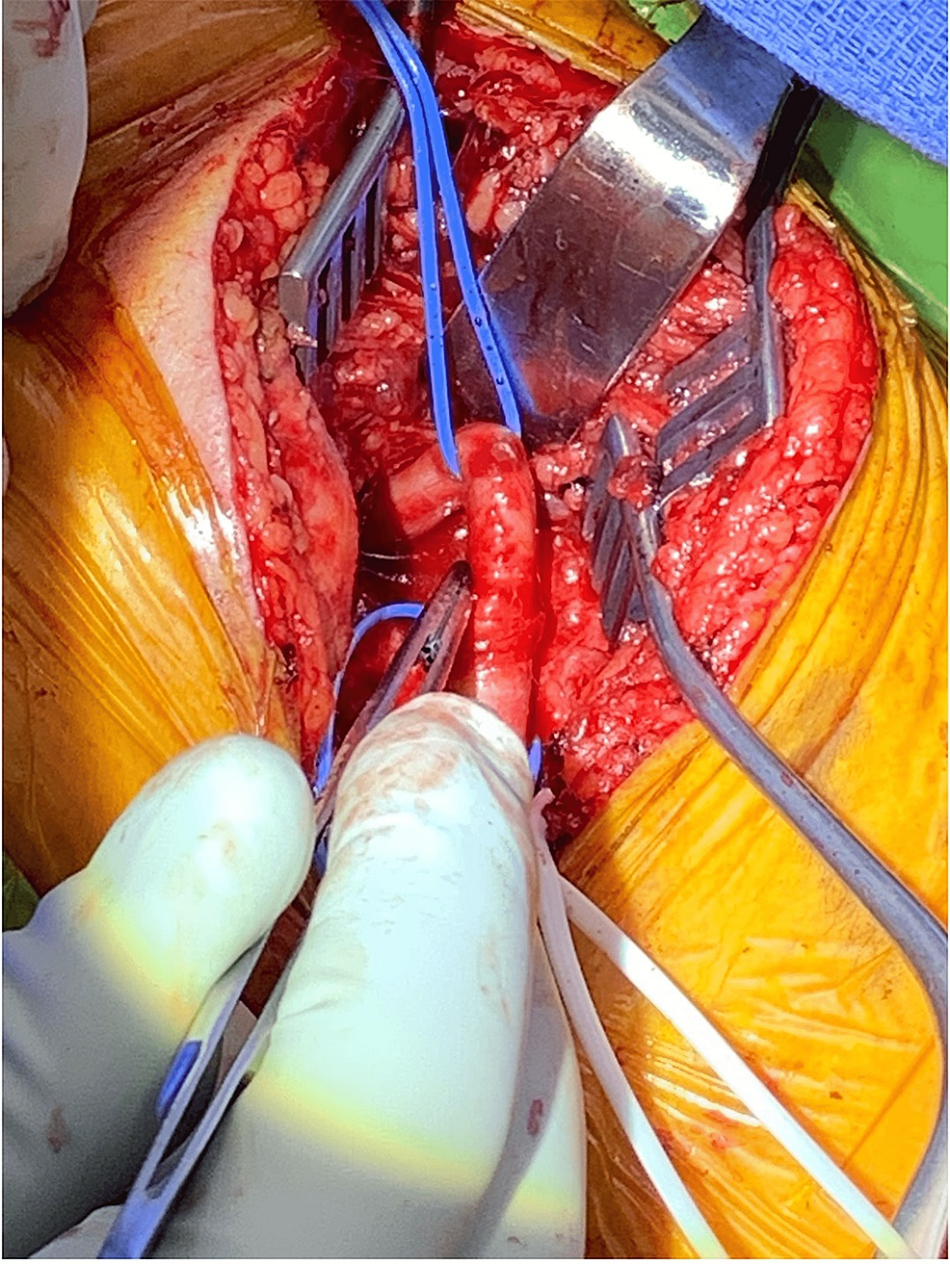
Intraoperative finding of Left ICA kinking. ICA: Internal carotid artery

After a multidisciplinary discussion, the patient underwent a 5 cm segmental resection of the kinked segment of the left ICA with end-to-end primary anastomosis (Figure 4). The operation was successful, with no neurologic deficits and improvement of symptoms.

## Discussion

Carotid artery tortuosity has been previously reported in the literature and is estimated to have an incidence of 18 to 34%. Tortuosity of the common carotid artery is significantly rarer than that of the internal carotid artery (prevalence of 10-25%), and their clinical implications and pathogenesis differ [3]. 

During embryological development, the third aortic arch forms the common carotid arteries and the proximal internal carotid arteries bilaterally. The descent of the heart into the thorax straightens these vessels. However, any developmental defect in this process can lead to tortuosity [4]. In addition, older patients or those with connective tissue disease more frequently exhibit this variant. Arterial connective tissue disease or degeneration may have a role in this pathology [3]. A tortuous CCA is commonly asymptomatic but can have clinical consequences. These include pulsating neck masses, dysphagia, and kinking, leading to syncope [5,6]. There is a possible association between transient ischemic attacks (TIAs) and CCA tortuosity, as seen in a case report of a patient with TIAs improved following surgical correction of this variant [4]. These variants must be considered before central venous catheter insertion and open surgical procedures such as tracheostomies. They may lead to complications such as hemorrhage if damaged [7]. Those with advanced age, female gender, hypercholesterolemia, obesity, atherosclerosis, hypertension, enlarged heart dimensions, Marfan syndrome, and fibromuscular dysplasia more frequently have this variant suggesting a possible association [3]. Our patient with right CCA tortuosity demonstrated many of these clinical risk factors. 

Tortuosity of internal carotid arteries can include elongation, kinking, or coiling. Hereditary factors, fibromuscular dysplasia, and atherosclerosis are associated with its development [2]. Classification schemes have been devised for the tortuosity of the ICA. The Weibel-Fields Classification system divides internal carotid artery tortuosity into three groups. Type 1 includes tortuosity, where arteries elongate into a “C,” “U,” or “S” shape. Type 2 includes looped or coiled vessels that spiral around an axis. Type 3 includes kinked vessels where arteries are twisted into a “V” shape [8]. 

The clinical implications of these variants include surgical complications such as fatal hemorrhage, internal jugular vein stenosis, dysphagia, and globus sensation [9,10]. A controversial issue is the effect of tortuosity on cerebrovascular blood flow. It has been postulated that the variant led to decreased cerebral blood flow and cognitive decline. Kinking can also lead to syncope when the neck is turned abruptly, as was seen in our patient [11]. Del Corso L et al. showed that patients with ICA abnormalities, including kinking, tortuosity, and coiling, have a low association with stroke or atherosclerotic vascular disease [12]. In the first case, how this kinking contributed to the CVA is uncertain. Knowledge of the anatomical variations of the ICA is essential for radiologists, anesthesiologists, and surgeons to decrease complications when performing any procedure in the involved area [10]. Mukherjee et al. reported that a 44% incidence of abdominal aortic aneurysm was discovered in patients with this variant. This leads to a possible association between the weakening of the aortic walls in an aneurysm and weakened walls in the carotids leading to tortuosity [13]. Hence, the incidental finding of carotid artery tortuosity is possibly related to abdominal aortic aneurysms and may prompt further screening if discovered incidentally. In our patient, multiple syncopal episodes were attributed to a kinked left internal carotid artery. This patient’s cerebrovascular insufficiency (CVI) symptoms may have been related to this. This case also illustrates the importance of considering vascular anomalies in diagnosing vertigo and syncope. It is thus crucial to include a vascular workup in a patient presenting with rotation-induced syncope.

A variation of the tortuous internal carotid artery, in which it is bilateral, is called the kissing carotid artery. The “kissing” internal carotid artery variant has been seen in 5-10% [1]. Internal carotid artery juxtaposition has various clinical manifestations, including a submucosal mass in the posterior pharyngeal wall, dysphagia, and glossopharyngeal neuralgia. The association between kissing carotids and cerebrovascular accidents is controversial [14]. Some cases are associated with pituitary disease and can complicate transsphenoidal surgery [15]. The right CCA was coiled, redundant, and tortuous in the first patient. It highlights how tortuosity can be incidental and mimic the appearance of kissing common carotids, which has not yet been described in the literature.

## Conclusions

The anatomical variants described in these cases highlight how they can either be asymptomatic and discovered incidentally or represent significant pathologies. Clinicians should also be aware of these variants when diagnosing and treating patients with syncope. Furthermore, our patients had multiple associated risk factors, including CVI and atherosclerosis, that could have led to tortuosity. Further investigation is required into how knowledge of these variants should affect patient management and the strength of their association with comorbid conditions.
